# Exploring the status of retail private drug shops in Bangladesh and action points for developing an accredited drug shop model: a facility based cross-sectional study

**DOI:** 10.1186/s40545-017-0108-8

**Published:** 2017-07-11

**Authors:** Syed Masud Ahmed, Nahitun Naher, Tarek Hossain, Lal Bahadur Rawal

**Affiliations:** 1 Centre of Excellence for Universal Health Coverage, James P Grant School of Public Health, BRAC University, 5th Floor, (Level-6), icddr,b Building, 68 Shahid Tajuddin Ahmed Sharani, Mohakhali, Dhaka, -1212 Bangladesh; 2 Health Systems and Population Studies Division, icddr,b, Dhaka, 1212 Bangladesh

**Keywords:** Informal sector, Retail drug shops, Drug shop salespersons, Irrational use of drugs, Bangladesh

## Abstract

**Background:**

The private retail drug shops market in Bangladesh is largely unregulated and unaccountable, giving rise to irrational use of drugs and high Out-of-pocket expenditure on health. These shops are served by salespersons with meagre or no formal training in dispensing.

**Method:**

This facility-based cross-sectional study was undertaken to investigate how the drug shops currently operate vis-a-vis the regulatory regime including dispensing practices of the salespersons, for identifying key action points to develop an accredited model for Bangladesh. About 90 rural and 21 urban retail drug shops from seven divisions were included in the survey. The salespersons were interviewed for relevant information, supplemented by qualitative data on perceptions of the catchment community as well as structured observation of client-provider interactions from a sub-sample.

**Results:**

In 76% of the shops, the owner and the salesperson was the same person, and >90% of these were located within 30 min walking distance from a public sector health facility. The licensing process was perceived to be a cumbersome, lengthy, and costly process. Shop visit by drug inspectors were brief, wasn’t structured, and not problem solving. Only 9% shops maintained a stock register and 10% a drug sales record. Overall, 65% clients visited drug shops without a prescription. Forty-nine percent of the salespersons had no formal training in dispensing and learned the trade through apprenticeship with fellow drug retailers (42%), relatives (18%), and village doctors (16%) etc. The catchment population of the drug shops mostly did not bother about dispensing training, drug shop licensing and buying drugs without prescription. Observed client-dispenser interactions were found to concentrate mainly on financial transaction, unless, the client pro-actively sought advice regarding the use of the drug.

**Conclusions:**

Majority of the drug shops studied are run by salespersons who have informal ‘training’ through apprenticeship. Visiting drug shops without a prescription, and dispensing without counseling unless pro-actively sought by the client, was very common. The existing process is discouraging for the shop owners to seek license, and the shop inspection visits are irregular, unstructured and punitive. These facts should be considered while designing an accredited model of drug shop for Bangladesh.

## Background

Retail drug shops are often the first and only source of healthcare outside home for a majority of patients in developing countries such as Bangladesh [1, 2]. It has been found in Bangladesh that more than 80% of the population seeks care from untrained or poorly trained *Pallichikitsoks* (village doctors) and drug shop retailers [3]. Currently in Bangladesh, 1,06,919 licensed [4] and an estimated equal number of unlicensed retail drug shops are involved in selling all types of drugs beside the “over-the-counter (OTC)” drugs. The latter are ‘medicines sold directly to a consumer without a prescription from a healthcare professional, as opposed to prescription drugs, which may only be sold to consumers possessing a valid prescription’ [5]. These are usually safe if used at recommended doses and duration, but can also be abused like other prescription drugs e.g., Cough medicines (e.g. Dextromethorphan), Cold medicines (e.g. Pseudoephedrine), Motion sickness pills (e.g. Dimenhydrinate), and Pain relievers (e.g. Acetaminophen). Bangladesh has currently 39 allopathic medicines in the OTC drug list [6]. About 2/3rd of the total health expenditure is from out-of-pocket (OOP), and of this, 65% is spent at the private drug retail shops [7].

According to law, the person dispensing drugs at drug retail outlets i.e., drug shops (drug shop salespersons, drug sellers) should at least have a ‘Grade C pharmacist’ certificate for the dispenser. This certificate is given after a short training of 12 weeks duration covering the basics of dispensing practice without any hands on training and is being conducted by the Pharmacy Council of Bangladesh (PCB) since 1995, in association with the Chemist and Druggist Association (shop owners association) of Bangladesh (BCDA) [8]. Only when this criterion is fulfilled, the shop owner can apply to the Directorate General of Drug Administration (DGDA) for issuance of a drug (pharmacy) license [9]. However, most of the salespersons at these retail drug shops do not have training in dispensing drugs or in diagnosing and treating medical conditions, which are the tasks they frequently perform [10]. Because drug shop salespersons have no other channel of information from the formal sectors open to them, they fall easy prey to the aggressive marketing strategies of the pharmaceutical companies [11]. Overprescribing, polypharmacy, prescribing unnecessary and expensive brand drugs, dispensing drugs without prescription, and OTC sell of antibiotics and steroids have been found to be the most common problems with these retailers [12–15].

Experiences in other parts of the world have demonstrated that private-sector drug seller initiatives designed on the basis of an accreditation and regulation model are feasible, improve access to medicines, and can be scaled up [16, 17]. Given the importance of the informal sector and private retail drug shops in Bangladesh, better regulation of the sector offers an opportunity to provide better pharmaceutical services which is essential for reducing total health expenditure and achieving universal health coverage in the country. There is dearth of data for informing the design of an accredited model of drug shops in Bangladesh. This study was undertaken to fill in this knowledge gaps by collecting relevant data on drug shops including its interactions with the regulatory regime; education, training and dispensing characteristics of the salespersons; users’ perceptions about the services provided by, and the technical knowledge of, the dispensers; and stakeholders’ experiences and opinions regarding the feasibility of designing such a model for Bangladesh.

## Methods

### Study aim and design

This was a facility-based (drug shops) cross-sectional study, exploring the status, mode of operation, and regulatory practices of the retail drug shops in the private sector in Bangladesh. It included samples of retail drug shops from rural and urban areas in all seven administrative divisions of the country.

### Settings

For the purpose of this study, the ‘drug shops’ were defined as private retail drug shops which are eligible for mandatory approval and registration from the DGDA under the Ministry of Health and Family Welfare of the Government of Bangladesh (MOHFW, GOB). This definition did not include facilities that sell Ayurveda, Homeopathy, and Unani remedies at retail shops.

A variety of methods was used to elicit relevant data: (a) document review (for data on policy and regulatory environment); (b) quantitative survey (for profiling retail drug shops, and gathering data on training and working experiences of the salespersons); (c) qualitative exploration (for eliciting perception of the catchment community and other stakeholders), and (d) structured observation in a subsample of the drug shops to document client-salesperson interactions in real time.

Respondents of the study included (a) the salesperson or the owner of each of the drug shops who was present at the time of the survey (in 12% of the cases where the owner was a different person, only the salespersons were interviewed); (b) a sample of respondents from the catchment areas of the shops who visited the nearest drug shop within past one month (for focus group discussions); (c) relevant stakeholders e.g., DGDA authorities at the district and central levels, and the representatives of drug related associations and experts (in-depth interview).

### Sampling strategy and sample

#### Quantitative survey

The rural samples included drug shops in the neighborhood of the *upazila* (sub-district) health complex (UHC), market places, and key business hubs. Because of time and resource constraints, we conveniently limited the number of sampled *upazilas* to 30 that were randomly selected from the seven administrative divisions, the number of *upazilas* being proportionate to the size of the division. Again, only one *upazila* was selected conveniently from each district in a division: either a *upazila* situated distally in a central district of a division or a *upazila* situated centrally in a peripheral district of the division. Next, three retail drug shops were selected from each of the sampled *upazila* which fulfilled the following criteria:

1. Drug shop in the neighborhood of the UHC, or within three kilometer radius of the UHC
2. Drug shop on the basis of their distance from the UHC (one nearest to the UHC, one farthest from the UHC, and one at a distance in-between) as available
3. Drug shop that had substantial customer visits (based on spot assessment)

The urban sampling was relatively simpler, taken exclusively from the seven city corporation areas (out of a total of 11 city corporations), which are the divisional headquarters. From the central thana (a police district, also known as a kotwali thana) of each city corporation area, three drug shops were selected using the following criteria (*n* = 21):

- First drug shop was selected from the central area near the kotwali thana
- Second drug shop was selected 1 km away from the first one
- Third drug shop was selected from the border area of the city (peri-urban area)

**Fig. 1:**
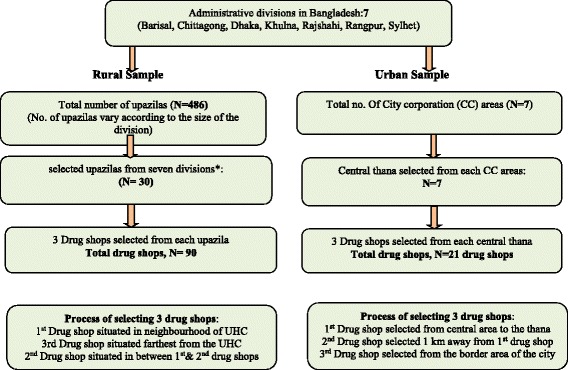
Flow chart describing sampling of drug shops in the rural & urban areas. *no. of upazillas selected for study according to their proportion in the different divisions (30/486)

**Fig. 2:**
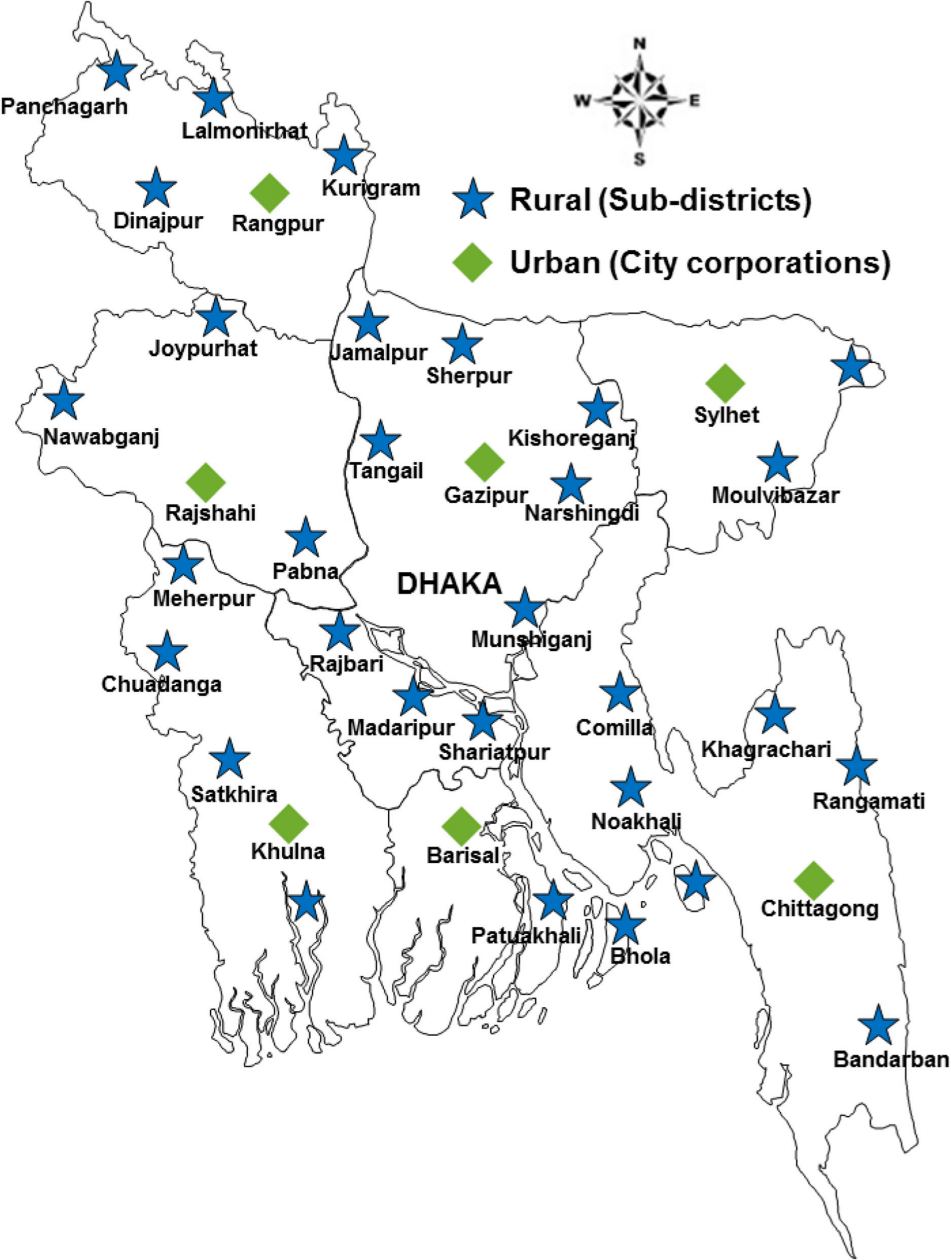
Rural and urban sites from where the sample upazilas (rural) and thanas (city corporation areas) were drawn. (Source http://www.ngof.org/nrc/wdb/watsanmap.php)

Thus, a total of 111 drug shops (90 rural and 21 urban areas) from the seven administrative divisions were included in the survey (see flow chart, Fig. 1). The sampled sites from where the sample upazillas/urban thanas were selected are shown in Fig. 2.

#### Qualitative exploration

##### Focus group discussions (FGD) and key informant interviews (KII)

For the FGDs, the catchment areas of a sub-sample of the surveyed shops at the divisional levels were selected. From these areas, a sample of patients/clients who have had visited the drug shops for buying medicines or other services within past 30 days from the day of survey were selected. In all, 16 such FGDs were conducted. Stakeholders from divisional head quarters (drug superintendants or drug inspectors working under the district DGDA) and high officials of the DGDA head quarter at Dhaka were conveniently selected for KI interviews (*n* = 22).

##### Structured observation

Structured observations of interaction between the salesperson and the patient/client visiting the 111 drug shops surveyed were done to document the nature and contents of the interaction. These were carried out simultaneously by a team of two interviewers: one proceeded with the survey while the other took notes about such interactions occurring during the survey. To reduce Hawthorne effect, interviewers spent some time for rapport building before the actual data collection took place. The observations documented were not specific to any particular illness but any such interaction that occurred during the period of survey. A total of 345 such interactions (three two hours session at different times of the day to capture the diversity) were documented. The study methods for addressing particular objectives, sanpling frame and sample size is shown in Table 1.

**Table 1:** Study methods and objectives addressed, sample Size, and sampling frame

Methods (objectives addressed)	Sampling frame
Drug shop survey (characteristics of the drug shops, salespersons, and regulatory environment) (*n* = 111)	Salesperson or dispensers from sampled retail drug shops
Structured observation of interaction between salesperson and clients (dispensing practices) (*n* = 345)	Interactions between salesperson or dispensers and clients visiting sampled retail drug shops
Focus group discussion (client perception of salespersons and experiences of services received from the drug shops) (*n* = 16)	Community members visiting the sampled retail drug shops
Key informant interview (stakeholders’ perceptions regarding current operation of drug shops and feasibility of designing an accredited model of drug shop fro Bangladesh) (For list, see appendix C)	i. Regulatory authority at district levels (drug superintendent or inspector) ii. National-level key stakeholders (from DGDA, BCDS,PCB)

*DGDA* Directorate General of Drug Administration, *BCDS* Bangladesh Chemist and Druggists Samity, *PCB* Pharmacy Council of Bangladesh

##### Tools development and training of data collectors

Tools for data collection such as the survey questionnaires (adapted after contextualizing from tools used by similar study in Tanzania) [18], Focus Group Discussions (FGD) and Key Informant Interviews (KII) and structured observation guidelines were developed and pre-tested for consistency, culture sensitiveness and ease of administration in the field before finalization.

The field data collectors (field interviewers) recruited for this study were science graduates with experiences in conducting community surveys and qualitative interviews in health sector. They underwent five days of intense training which consisted of didactic lectures on the content of the instruments followed, mock interviews, and field-testing through repeated practice sessions outside the study areas. End-of-training debriefings and necessary feedback, suggestions, and guidance were provided by the research team. Seven teams were formed, each with four to five interviewers and a team leader.

##### Data collection and quality control

The day-to-day field activities of the teams (total seven) were overseen by the team leaders and assigned supervisors. To ensure completeness of the survey, the field research team members cross-checked each other’s filled-in questionnaires every day before leaving the survey sites under the supervision of the team leader. The whole survey activity was supervised and managed by the PIs and co-PIs, who made frequent field visits and provided assistance and guidance as and when needed.

An independent, mobile quality-control team also randomly checked 5%of the questionnaires completed the previous day in the working areas of each of the study teams. Such checks occurred in random spots in the field and feedbacks were provided to the team on any errors or gaps identified. Instructions for corrections were communicated to all the teams simultaneously to ensure standardization.

Again, the research team in the central office also cross-checked all questionnaires for inconsistencies not conforming to the instructions given at the time of training of the interviewers. If any such error was found, the questionnaire was discarded, and the survey was redone. This extensive process ensured the quality of data in a time of great political instability and difficulty of movement in the field between January and February 2015.

##### Data management and analysis

The field activity and data entry work was completed within 45 working days. When the field activities were completed, data were entered using the statistical package for the social sciences (SPSS version no. 17) by a professional data management team. Of the total data, 5% was re-entered to control data-entry quality. During the data entry, the PIs and project manager supervised the data management team to ensure the quality and validity of the data, and the codebook developed by the research team was used for this purpose. When data entry was completed, the files were transferred into the SPSS for cleaning, consistency check, and cross-tabulation. The research team analyzed the final cleaned dataset using SPSS, following an analysis plan prepared beforehand.

For the qualitative data, content analysis was done with consolidation of relevant codes into sub-themes and themes. For example, some of the important themes included drug shop licensing (sub themes: licensing process, drug shop owners’ perception of the process, informal payment for license, capacity of the regulatory authority etc.), dispensing practices (sub themes: capacity of the salespersons, selling non-OTC drugs without prescriptions, counseling etc.), and accredited drug shop model (sub themes: necessity and requirements, stakeholders’ perceptions, services to offer etc.). Triangulation of data was done by eliciting information on a particular issue from different sources e.g., Observation data on shop visits by the clients without prescription was cross-checked in FGDs with community members in the catchment areas of the shops as well as stakeholders such as service providers and regulatory personnel.

## Results

### Findings from the quantitative survey

The quantitative survey involved 111 drug shops in total, 90 from rural areas and 21 from urban areas. Majority of the drug shops (45%) were running for more than 10 years, in greater proportion in the rural areas (Table 2).

**Table 2:** Characteristics of the sampled drug shops %

	Rural	Urban	All
	(*n* = 90)	(*n* = 21)	(*N* = 111)
Drug shop has been in operation for_			
< 5 years	32	38	33
5–10 years	21	24	22
10+ years	47	38	45
Distance from nearest health facility is ≤30 min walk	90	95	91
Possess drug license (reported)	81	90	83
Possess trade license (reported)	96	100	96
The shop was reported to be inspected by a Drug Inspector			
Within past one year	84	79	83
Within past two years	16	21	17
Stock register maintained (reported)	8	14	9
Drug sales record maintained (reported)	8	19	10
Functional refrigerator available (checked)	31	33	31
Besides dispensing, additional services available (multiple response)			
Pushing injections	59	67	60
Diagnostic services (e.g., testing blood sugar, measuring blood pressure)	62	67	63
Vaccination services (e.g., TT, rabies vaccine)	30	38	31
Burn and wound treatment/dressing	66	52	63
Source of clients (multiple responses)			
Public health facilities/clinics/hospitals	34	19	31
Private health facilities/clinics/hospitals	10	19	12
Private chambers of doctors	7	5	6
Self-referral	67	76	68
Clients visiting daily without prescription^a^	69 (*n* = 4511)	64 (*n* = 973)	65 (*n* = 5484)

^a^average number of clients visiting a drug shop in a day as reported by the respondents

Interestingly, more than 90% of the drug shops were physically located within 30 min walking distance of some health facilities, mostly in the public sector. Most (96%) of the shops reportedly possessed trade license, but a lesser proportion (83%) reported to have a drug license. However, the validity of the drug licenses was not checked in this survey. Within past one year, 83% of the shops were reported to have been inspected at least once by a drug inspector from the local (district) DGDA office.

Sixty-five percent of the visiting clients (presumably, out of an average tentative number of clients visiting in a day) came to purchase drugs without a prescription (Table 2). The clients visited the shops mostly by self-referral which was more common in the urban shops (76%) compared to the rural shops (67%). Besides dispensing drugs, the shops also provided some additional services such as pushing injections (60%), some petty diagnostic services such as measuring blood pressure or testing for blood sugar (63%), first-aid services such as wound and/or burn dressing (63%) etc. Only 9% of the shops reported to have maintaineda stock register while 10% maintained a record of drugs sold (Table 2). Around 1/3rd of the shops surveyed were found to have a functioning refrigerator.

In 76% of cases, the owner and the salesperson was the same person, more so in the urban shops (Table 3). Forty-nine percent of them had no formal training in pharmacy/dispensing and of those who had, the overwhelming majority (93%) had a three months training in a Grade C Certificate Course only. Majority of those receiving ﻿some ad-hoc training received it from the pharmaceutical companies (38%). The other group i.e., without any training whatsoever, learned the trade through apprenticeship with fellow drug retailer (42%), relatives (18%), village doctors (16%) and so on (Table 3).The respondents valued the necessity of training for a variety of reasons such as efficient dispensing (66%), gaining knowledge about rules and regulation of dispensing (84%), and developing good relation with clients and goodwill for business (21%).

**Table 3:** Characteristics of the salespersons attending the sampled drug shops

	Rural	Urban	All
	(*n* = 90)	(*n* = 21)	(*N* = 111)
*Salesperson and owner is the same person*	76	81	76
*Educational status of the salespersons*			
≤ 10 yrs. of schooling	4	—	4
10 completed years of schooling (SSC)	27	38	29
12 completed years of schooling (HSC)	32	38	33
Graduate	37	24	34
*Involvement in any other occupation*			
Farming	48	20	43
Self-employment	15	40	19
Others^a^	37	40	38
*Pharmacy training status of the salespersons*			
Have formal training	49	62	51
No formal/some ad-hoc training	51	38	49
*Of those who have formal training,*	(*n* = 44)	(*n* = 13)	(*n* = 57)
Category B pharmacist training (one year Diploma in pharmacy)	7	8	7
Category C pharmacist training (three months Certificate course in dispensing/pharmacy)	93	92	93
*Of those who have some* ad-hoc *training, training is provided by_*	(*n* = 46)	(*n* = 8)	(*n* = 54)
Government organization	36	—	31
Private organization	29	—	25
Pharmaceutical companies	28	100	38
NGOs	7	—	6
*Of those receiving no training of any type, mode of informal learning (multiple responses)*	(*n* = 32)	(*n* = 6)	(*n* = 38)
Fellow drug seller in other store(s)	44	33	42
MBBS doctor in the drug shop or pharmacy^a^	9	17	10
Pharmacist	3	—	3
Village doctor	16	17	16
Relatives	22	—	18
LMAF course	3	—	3
Pharmaceutical company	3	33	8

*N* all observations, *n* observation subset, — not applicable, *SSC* Secondary School Certificate, *HSC* Higher Secondary Certificate

^a^nursery, fishery, business, or student

### Findings from qualitative exploration (presented by themes**)**

#### Drug shops, salespersons and dispensing practices

The drug shops were quite a familiar entity in the community life. As some of the drug shops were as old as 10 to 20 years, many remembered their interaction with the shops since their childhood. The salespersons of the drug shops were also a very common face to them as they mostly originated from the local community, were amiable and trustworthy, and well respected. Some clients (respondents from the catchment community who purchased drugs from the sampled shops within past one month) mentioned that they had a very good relation with the people at the drug shops.

The clients were aware that the salespersons may have some training on drug dispensing but in most instances, they did not bother much. Some even said that training was not necessary as they already have many years of experience. To quote one:

“He doesn’t need training; he already has achieved vast knowledge on drug dispensing from his 30 years of work experience.”

-A drug shop client from Dhaka

However, some emphasized the importance of education and training in dispensing:

*“…if the drug seller does not know about drug dispensing, he cannot provide me proper medicine that I need…”*

“…if the drug seller has educational and professional qualification, then he will be able to provide us a proper service…”

-A drug shop client from Khulna

The need of prescription for buying drugs other than the’ Over-the-counter (OTC) drugs’ was considered superfluous by the respondents as the salespersons were known to them. They explained that if a person required a drug, s/he would explain his/her illnesses to the salesperson, and get necessary drugs based on symptoms. Structured observation done on a number of ‘client-salesperson interactions’ at drug shops also corroborated this process. For example, in about one-third of such interactions in the urban areas, only 36% salespersons asked about the nature of illness, 27% about the symptoms of illness, and 26% about the duration of the illness. Sometimes, depending upon symptoms, the salesperson made a superficial, nonspecific physical examination such as feeling the client’s pulse, checking for anemia, measuring temperature and blood pressure, auscultation of the chest with a stethoscope if there is cough, pressing the abdomen if there is a complaint of digestive disturbances etc.

Clients having chronic illnesses also didn’t think it necessary to show the prescription when buying the particular drug. They usually used to bring old receipts or empty medicine packets to buy the same drug again. They were also complacent about receiving receipts against the cash paid for drugs, the transactions taking place based on mutual trust:

“The drug seller does not provide any money receipt and, as a consumer, I do not feel needs of collecting the money receipt because we both know each other and we are neighbors.”

-A drug shop client from Chittagong

Most respondents said that the salespersons explained to them about the quantity, frequency, time and duration of intake, whether to take the medicines before or after a meal, and how to prepare syrup for kids and so forth. To quote:

“Sometimes the drug seller counsels us about the total doses that if you do not complete your dose … you will not get effective result.”

-A drug shop client from Khulna

If a prescribed drug is not available, the drug sellers often would substitute another brand, sometimes without informing the client. For situations which the drug seller could not manage after repeated attempts, patients are usually referred to a doctor or a nearby health facility. One respondent reported:

“If the patient’s condition became worse than his previous condition, then the drug seller usually changes the medicine.”

-A drug shop client from Rangpur

### Regulatory processes

According to the law, the storage, display, and sale of drugs are punishable crimes without a license issued by the DGDA. At the time of the study, the DGDA had licensed 1,15,439 drug shops, but it was suspected that there are an approximately equal number of shops which are unlicensed. Because of a shortage of required personnel, the inspection and checking process of the drug shops appeared to be superficial, few and far between, and that process was not compliant with stipulated laws. One drug inspector summarized the field-level problems of inspection:

“We don’t have our own office, no transport facilities, no protocol.There are around 3,000 shops in my area! Civil surgeon of the hospital… has car facilities; my work area is more, but I don’t have that facility”

-A drug inspector

Interestingly, many respondents did not know that a license is required before opening a drug shop. To quote one respondent:

“I do not know the importance to have a license for a drug shop…”

-A drug shop client from Chittagong

Yet another emphasized its importance:

“…I think it is a good practice to have a license because if they have a valid license …they will not be able to dispense harmful medicine.”

-A drug shop client from Barisal

However, the respondents perceived the process of applying for and getting a drug license to be a cumbersome, lengthy, and costly process. Resentments were echoed uniformly by the owners/salespersons regarding both the licensing and the inspection process. All tiers of the regulators were of the opinion that the existing system is inadequate. One inspector observed:

“Once I observed that the drug seller shut down his shop, while the government authority came to inspect the drug shops of this area.”

-A drug inspector from Rangpur

Key-informant interviews with field-level drug inspectors emphasized that the existing regulatory system needed to be improved in areas such as logistics, availability of trained personnel, adequate budget for regular and timely inspection visits, and reduction of lead time for issuing the license after application.

The numbers of unlicensed shops are large, and the Bangladesh Chemists and Druggists Samity (i.e., Association, BCDA)thought this situation to be due to problems with the regulatory authority’s capacity to oversee the market. They complained that rules and regulations are frequently not enforced, resulting from corruption at the grassroots level. As BCDA does not have a monitoring role, the association informs the relevant DGDA authorities about irregularities that come to their notice. The BCDA informed that it has distributed about 200,000 informational leaflets to build awareness among the consumers. To address the severe shortage of trained dispensers, the BCDA, with technical help from the Pharmacy Council of Bangladesh (PCB) conducts a three-month long training course to award grade C certificate for dispensers working in the drug shops.

### Feasibility of developing an accredited model of drug shops for Bangladesh

The regulators at the central and district levels were unanimous about the necessity of developing an accredited model of a drug shop (Accredited Drug Shops, ADS) in the country to improve the current chaotic situation. According to the regulators, this is all the more necessary because Bangladesh has a large illiterate semiliterate population who can’t distinguish the good from the bad. Because a drug shop is the first contact with any kind of health care service for a majority of the people, especially those in the rural and remote areas, this accreditation is a must for maintaining standards and quality. The regulators at the DGDA preferred the model drug shops to be run by the registered pharmacists i.e., those who are graduates or have diplomas. As one respondent from the DGDA explained:

“Pharmacists should dispense medicine to patients as per doctor’s prescription. If any doctor writes a wrong medicine, the pharmacist should consult with the doctor before giving the medicine to the patient. Pharmacists will counsel the patient how to take medicine and will explain this properly. A pharmacist will check the expiry dates of the medicines in the shop … he will ensure whether the shop is well-organized, and the records are well-maintained.”

-A regulatory authority from Barisal

However, in the short term, these could be run by at least a person certified in the practice of dispensing. They also suggested extensive review of the current course for content and pedagogy including recruitment of students from science background, inclusion of topics in the curricula such as Primary Health Care (PHC), first aid, legal and ethical aspects of dispensing, inter-personal communication with customers, pharmacist and customer relationship and rational use of drugs. In addition, they advised for some job placement or in-service training or practical experiences before the pharmacist certificate can be issued. A number of suggestions were put forward e.g., organizing a “Drug Day” or “Drug Week” campaigns and arranging a “Drug Fair” to generate awareness among the community and drug sellers/owners.

## Discussion

This study provides necessary information for developing of an accredited model of drug shops in Bangladesh by focusing on (a) consumer perceptions about the drug shops and its salespersons, (b) regulatory regime, and (c) the challenges and opportunities for designing an accredited drug shop model for Bangladesh. The findings and its implications are discussed under these themes, and finally, some recommendations are made how to go forward with pilot testing such a model in near future.

### Consumer perception about drug shops and salespersons

Most community members were not concerned about the educational and technical credentials of the drug sellers/dispensers and considered the salespersons/dispensers as a “friend in deed.” This view is very common in low- and middle-income countries (LMICs), especially in the remote and hard-to-reach areas that do not have doctors or, for that matter, any formal health facilities or providers [12, 13, 16, 19–22]. Purchasing non-OTC drugs without a prescription is also common in Pakistan [23], India [24] and other LMICs [25] as it is in Bangladesh. When seeking treatment advice by the patients/clients by describing symptoms, the practice of arriving at a diagnosis by a brief history taking and nominal physical examination, is all too common with drug sellers in the LMICs [25].Lack of professional training of any kind, as observed in this study for nearly half of the respondents, is not uncommon in countries such as the Lao People’s Democratic Republic, Pakistan, and Vietnam [12, 23, 26].

### Regulatory regime

It appears that the lengthy and costly licensing process discouraged the drug shop owners from applying for a license. One reason identified by the shop owners is that the license application, after field inspection by the Drug Inspectors/Superintendent, has to be placed before the district drug committee for approval. Only then, it can be sent to DGDA office, Dhaka for approval. Further inquiry revealed that the district committee meetings are few and far between and this result in backlogs. Second, the alleged informal costs associated with getting a license over and above the required government fees discourage the unlicensed drug shops to get registered. Third, due to constraints in human and financial resources of the regulatory authority which is critical for regulation [27], the shop visits are not regular and systematic too. The resulting short time allocated to a visit allow the inspectors and superintendents to have a superficial inspection only which is not helpful for enforcing regulation, a common problem with enforcing regulation in general in the broader health system [28]. Thus, the creation of a user-friendly regulatory environment is essential for improving the pharmacy practices at the drug shops, including the unlicensed shops [25]. The critical role of Consumer Association of Bangladesh (CAB) and similar associations should be leveraged to promote the rational use of drugs.

### Challenges and opportunities for developing an accredited drug shop model for Bangladesh

The Accredited Drug Shop (ADS) model of retail drug shops such as tested in Tanzania standardizes the drug shop’s operation by staffing the shop with appropriately trained dispensers, strictly enforcing regulations, and providing supportive supervision [18]. The current state of drug shops in the private informal sector in Bangladesh justifies an adapted model for Bangladesh, and is feasible as revealed by different stakeholders in this sector. However, for the model’s implementation, certain conditions have to be fulfilled as elicited from the regulators and other stakeholders using qualitative methods. These include, but are not restricted to (1) training a cadre of dispensers/community pharmacists using a revised and updated curriculum, (2) franchising the training of vast numbers of salespersons/attendants at drug retail outlets to registered training organisations/institutions as a stop gap measure, (3) expanding pharmacy services beyond dispensing drugs (e.g., first-aid services, measuring and monitoring blood pressure and blood sugar for diabetes, and provision of some basic curative services as provided by the Community Health Workers (CHWs), and (4) building community awareness on the importance of quality pharmacy services and rational use of drugs.

### Study Limitations

Though the salespersons were asked whether their drug shop possessed drug and trade license but due to sensitive nature of the issue, these could not be physically verified. The sample size could have been expanded and made more representative to capture local level variations but was limited by time and resource constraints.

#### So, how to overcome the ground realities for designing an ADS model: **Recommendations**

To summarise, Bangladesh has now a large number of private drug shops where all kind of drugs are sold by salesperson with meagre or no training in dispensing whatsoever, under the supervision of a weak and poorly resourced regulatory body. To overcome this chaotic scenario, the following measures are suggested:

i. In the immediate, **short term (one to three years**): To restore some discipline in the prevailing chaotic situation and bring the informal drug shops under some regulation, the drug licensing process should be made user-friendly, less time consuming, efficient, and inexpensive so that the shop owners are encouraged to become licensed after fulfilling the requirements. The inspection visits to drug shops should be regular, comprehensive, supervised (by district DGDA), and problem solving—not punitive. This would ensure enforcement of minimum regulatory requirements. Finally, some expedited measures (‘crash programme’) need to be in place to train the vast number of drug sellers/salespersons in unlicensed shops within as short a time as practically feasible. To accomplish this, public-private partnerships (PPPs) including non-profit Non-Government-Organization (NGOs) should be encouraged to play a significant role in franchising the above Grade C Certificate (course for dispensers) course.
ii. **In the long term** *,* three basic tasks should be prioritized. *First,* re-visiting the current curriculum and revising the contents, forms, and duration of the grade C certificate course for the dispensers, using modern pedagogic methods. It should also have an in-built internship programme where they would receive hands-on training by working under a qualified pharmacist for a fixed period of time. The curriculum may include basic PHC services (standard for CHWs) so that it can serve as the first health post in remote and hard-to-reach areas.

*Second,* all efforts should be made to increase the human and technical capacity of the DGDA (e.g., establish more drug-testing laboratories) to streamline regular inspection visits of the drug shops and discourage marketing of counterfeit, expired, and poor-quality drugs. Possibility for establishing the digital pharmaceuticals systems should also be explored mainly for the purpose of registration and renewal of drug shop licence, verifying the genuine drug products and facilitating effective monitoring systems.

*Third,* to regulate the unethical and aggressive marketing practices of the pharmaceutical companies and their medical representatives [11], and promote rational use of drugs, measures should be taken to enforce the ‘Code of Pharmaceutical Marketing Practices’ promulgated in 1994 [29]. Last but not the least, an extensive information, education, and communication (IEC) campaign to build awareness of the public and pharmacy stakeholders on the irrational use of drugs and their harmful effects (e.g., antimicrobial resistance, MDR TB, etc.) should be launched.

## Conclusion

The private drug shop market in Bangladesh is now largely unregulated and unaccountable, and run by salespersons who are mostly trained informally through a process of ‘apprenticeship’. Clients visiting drug shops without a prescription, and inadequate dispensing practice resulting in OTC sale of all forms of medicine was very common. The existing process is discouraging and time consuming for the shop owners to seek license, and the shop inspection visits are irregular, unstructured and punitive. Urgent measures such as a ‘crash’ programme to train the salespersons; making inspection visits regular, comprehensive, and problem solving; improving technical and human resource capacity of the regulatory authority and making the licensing process ‘user-friendly’ are needed to improve the current chaotic scenario and mainstream these shops into the existing PHC infrastructure. Besides, key action points for designing an accredited drug shop model for Bangladesh is discussed informed by these findings.

## Abbreviations

ADS: Accredited Drug Shops
AHUB: Ayurveda, Homeopathy, and Unani Board
BCDA: Bangladesh Chemists and Druggists Samity
CHWs: Community Health Workers
DGDA: Directorate General of Drug Administration
FGD: Focus Group Discussions
GOB: Government of Bangladesh
HRH: Human Resource for Health
IEC: Information, Education and Communication
KII: Key Informant Interviews
LMICs: Low- and Middle-Income Countries
MDR TB: Multi-Drug-Resistant Tuberculosis
MOHFW: Ministry of Health and Family Welfare\
NGOs: Non-Governmental Organization
OOP: Out-Of-Pocke
OTC: Over-the-counter
PCB: Pharmacy Council of Bangladesh
PHC: Primary Health Care
PPPs: Public-Private Partnership
SPSS: Statistical Package for the Social Sciences
UHC: Upazila Health Complex

## References

[1] Ahmed SM, Hossain MA, Chowdhury MR (2009). Informal sector providers in Bangladesh: how equipped are they to provide rational health care?. Health Policy Plan.

[2] Ahmed SM, Hossain MA, Chowdhury AMR, Bhuiya AU (2011). The health workforce crisis in Bangladesh: shortage, inappropriate skill-mix, and inequitable distribution. Hum Resour Health.

[3] Ahmed SM, Hossain MA (2007). Knowledge and practice of unqualified and semi-qualified allopathic providers in rural Bangladesh: implications for the HRH problem. Health Policy.

[4] Directorate General of Drug Administration DGDA website. http://www.dgda.gov.bd/. Accessed 8 June 2017.

[5] Over-the-counter drugs. https://en.wikipedia.org/wiki/Over-the-counter_drug*.* Accessed 7 June 2017.

[6] List of allopathic OTC drugs. http://dgda.gov.bd/index.php/laws-and-policies/204-national-drug-policy-2016-including-essential-drug-list-and-otc-list/file Accessed 7 June 2017.

[7] Ministry of Health and Family Welfare, Government of Bangladesh (MoHFW, GoB) (2015). Bangladesh National Health Accounts 1997–2012.

[8] Mazid MA, Rashid MA. Pharmacy education and carrier opportunities for pharmacists in Bangladesh. Bangladesh Pharm J. 2011;14(1):1-9.

[9] Ministry of Law, Government of Bangladesh. The Pharmacy Ordinance 1976 (Ordinance No. XIII of 1976). http://bdlaws.minlaw.gov.bd/print_sections_all.php?id=513. Accessed 8 June 2017.

[10] Amzad R (2012). To explore the knowledge and practices of the drug shop attendants regarding dispensing and prescribing (disease specific) medicines and the difference, if any, between the ‘grade C’ pharmacy certificate holders and the other (those who don’t have grace C certificate) in these aspects. MPH Dissertation.

[11] Mohiuddin M, Rashid SF, Shuvro MI, Naher N, Ahmed SM (2015). Qualitative insights into promotion of pharmaceutical products in Bangladesh: how ethical are the practices?. BMC Medial Ethics.

[12] Chuc N (2002). Towards good pharmacy practice in hanoi: a multi-intervention study in private sector.

[13] Chalker JC (2003). Interventions for improved prescribing and dispensing in Nepal, Thailand, and Vietnam.

[14] Chowdhury PM (2010). An overview of the pharmaceutical sector in Bangladesh.

[15] Bloom G, Wilkinson A, Tomson G, et al. Addressing resistance to antibiotics in pluralistic health systems. In: STEPS Working Paper. Brighton, STEPS Centre; 2015. p. 84.

[16] Goodman C, Brieger W, Unwin A (2007). Medicine sellers and malaria treatment in sub-Saharan Africa: what do they do and how can their practice be improved*?*. Am J Trop Med Hyg.

[17] Drug Seller Initiatives (DSI). Sustainable drug seller initiatives .2014. http://www.drugsellerinitiatives.org/about/projects/. Accessed 10 Sept. 2015.

[18] Rutta E, Liana J, Johnson K (2015). Erratum to: Accrediting retail drug shops to strengthen Tanzania’s public health system: an ADDO case study. BMC J Pharm Policy Practice.

[19] Kamat VR, Nichter M (1998). Pharmacies, self-medication, and pharmaceutical marketing in Bombay. India Soc Sci Med.

[20] Kafle KK, Madden JM, Shreshta AD (1999). Can Licensed Drug Sellers Contribute to Safe Motherhood? A Survey of the Treatment of Pregnancy-Related Anaemia in Nepal. Soc Sci Med.

[21] Stanback J, Otterness C, Bekita M (2011). Injected with controversy: sales and administration of injectable contraceptives in drug shops in Uganda. Int Perspect Sex Reprod Health.

[22] Wafula FN, Miriti EM, Goodman CA (2012). Examining characteristics, knowledge, and regulatory practices of specialized drug shops in sub-saharan africa: a systematic review of the literature. BMC Health Serv Res.

[23] Rabbani F, Cheema FH, Talati NS (2001). Behind the counter: pharmacies and dispensing patterns of pharmacy attendants in Karachi. J Pakistan Med Assoc.

[24] Sabde YD, Diwan V, Saraf VS (2011). Mapping private pharmacies and their characteristics in Ujjain District, Central India. BMC Health Serv Res.

[25] Miller R, Goodman C (2016). Performance of retail pharmacies in low- and middle-income Asian settings: a systematic review. Health Policy Plan.

[26] Stenson B, Syhakhang L, Eriksson B, Tomson G (2001). Real world pharmacy: assessing the quality of private pharmacy practice in the Lao People’s Democratic Republic. Soc Sci Med.

[27] Sauwakon R, Wondemagegnehu E. Effective drug regulation. A multicountry study. World Health Organization. 2002.

[28] Garimella S, Sheikh K. Workforce governance: reflections on the role of postings and transfers at the primary health care level. BMJ Open. 2015; 5 (Suppl 1):A1-53.

[29] Code of Pharmaceutical Marketing Practices. Ministry of Health and Family Welfare. Government of Peoples Republic of Bangladesh. http://www.dgda.gov.bd/index.php/2013-03-31-05-16-29/forms/77-code-of-pharamaceutical-marketion-practices/file. Accessed 8 June 2017.

